# LeNRT1.1 Improves Nitrate Uptake in Grafted Tomato Plants under High Nitrogen Demand

**DOI:** 10.3390/ijms19123921

**Published:** 2018-12-07

**Authors:** Francisco Albornoz, Marlene Gebauer, Carlos Ponce, Ricardo A. Cabeza

**Affiliations:** 1 Departamento de Ciencias Vegetales, Facultad de Agronomía e Ingeniería Forestal, Pontificia Universidad Católica de Chile, Avenida Vicuña Mackenna 4860, Macul, Santiago 7820436, Chile; mgebauer@uc.cl (M.G.); ctponce@uc.cl (C.P.); 2 Centro UC Desierto de Atacama, Pontificia Universidad Católica de Chile, Avenida Vicuña Mackenna 4860, Macul, Santiago 7820436, Chile; 3 Departamento de Producción Agrícola, Facultad de Ciencia Agrarias, Universidad de Talca, Avenida Lircay S/N, P.O. Box 747, Talca 3462227, Chile; rcabeza@utalca.cl

**Keywords:** nitrate transporters expression, growth rate, root membrane transporters, root respiration

## Abstract

Grafting has become a common practice among tomato growers to obtain vigorous plants. These plants present a substantial increase in nitrogen (N) uptake from the root zone. However, the mechanisms involved in this higher uptake capacity have not been investigated. To elucidate whether the increase in N uptake in grafted tomato plants under high N demand conditions is related to the functioning of low- (high capacity) or high-affinity (low capacity) root plasma membrane transporters, a series of experiments were conducted. Plants grafted onto a vigorous rootstock, as well as ungrafted and homograft plants, were exposed to two radiation levels (400 and 800 µmol m^−2^ s^−1^). We assessed root plasma membrane nitrate transporters (*LeNRT1.1, LeNRT1.2, LeNRT2.1, LeNRT2.2* and *LeNRT2.3*) expression, Michaelis‒Menten kinetics parameters (*V*_max_ and *K*_m_), root and leaf nitrate reductase activity, and root respiration rates. The majority of nitrate uptake is mediated by *LeNRT1.1* and *LeNRT1.2* in grafted and ungrafted plants. Under high N demand conditions, vigorous rootstocks show similar levels of expression for *LeNRT1.1* and *LeNRT1.2*, whereas ungrafted plants present a higher expression of *LeNRT1.2*. No differences in the uptake capacity (evaluated as *V*_max_), root respiration rates, or root nitrate assimilation capacity were found among treatments.

## 1. Introduction

The use of grafted plants has become a common practice among tomato growers, mainly because of the search for methods that enhance crop resistance to soil-borne pathogens [1]. Grafting usually results in vigorous plants with higher total and commercial yield than non-grafted plants [2]. In order to sustain this higher productivity, plants must absorb larger amounts of nutrients or use them in a more efficient fashion than ungrafted plants [3]. Identifying the mechanisms involved in higher uptake capacity or higher use efficiency of nutrients, especially nitrogen (N), will allow us to design more sustainable production systems by reducing the application of fertilizers, thereby limiting damage to the environment.

Nitrogen can be absorbed by plant roots either as nitrate (NO_3_^−^) or ammonium (NH_4_^+^); where tomato has a marked preference for absorbing NO_3_^−^ [4,5]. Five root plasma membrane NO_3_^−^ transporters have been identified in the tomato, belonging to the NRT1 and NRT2 families [6]. Two genes (*LeNRT1.1* and *LeNRT1.2*) comprise the LeNRT1 family, which encode for high-capacity, low-affinity nitrate transporters [7]. On the other hand, the NRT2 family comprises three genes (*LeNRT2.1*, *LeNRT2.2* and *LeNRT2.3*) encoding for low-capacity, high-affinity transporters [6]. The affinity for the uptake of a particular ion is described by the application of the Michaelis‒Menten relation to data obtained from depletion experiments [8]. In this experiment, plants are subjected to different concentrations of the ion of interest, and sequential sampling at regular intervals allows for the determination of uptake rates. Then, the uptake rate (*U*) and the external concentration (*C*) data are fitted to the following equation:

*U* = (*V*_max_ × (*C* − *C*_min_))/(*K*_m_ + *C* − *C*_min_), (1)

where *V*_max_ is the apparent maximum uptake capacity, *K*_m_ is the apparent affinity for the ion, and *C*_min_ represents the minimum concentration required for uptake to occur. High *V*_max_ values imply roots with enhanced uptake capacity, while low *K*_m_ values denote roots with high affinity for the ion [9].

The synthesis of these transporters in the roots depends on external NO_3_^−^ availability, but fluctuations in the expression levels have been reported according to the rate of shoot carbohydrate export to the roots or feedback regulation from N assimilates [7,10]. Plant internal N status regulates NO_3_^−^ uptake capacity [10], with both processes partly controlled by the activity of the enzymes involved in the assimilatory pathway. The first enzyme acting on NO_3_^−^ assimilation is nitrate reductase (NR), and the evidence in tomatoes suggests that it is exclusively localized in the leaves [11]. 

Plant N demand is ultimately controlled by the growth rate, and environmental factors such as light intensity determine biomass accumulation [12]. Therefore, the aim of this study was to assess the metabolic adaptations involved in root NO_3_^−^ uptake between grafted and ungrafted tomato plants subjected to different growth rates. The study was conducted with the hypothesis that vigorous rootstocks modify their root metabolism in order to absorb higher NO_3_^−^ quantities than ungrafted plants when required by plant N demand. The study was conducted using *Solanum lycopersicum* L. cv. Attiya (AT) and *S. lycopersicum* x *S. habrochaites* cv. Kaiser (KA) as the rootstock material.

## 2. Results

### 2.1. RGR and Plant N Uptake

**Figure 1:**
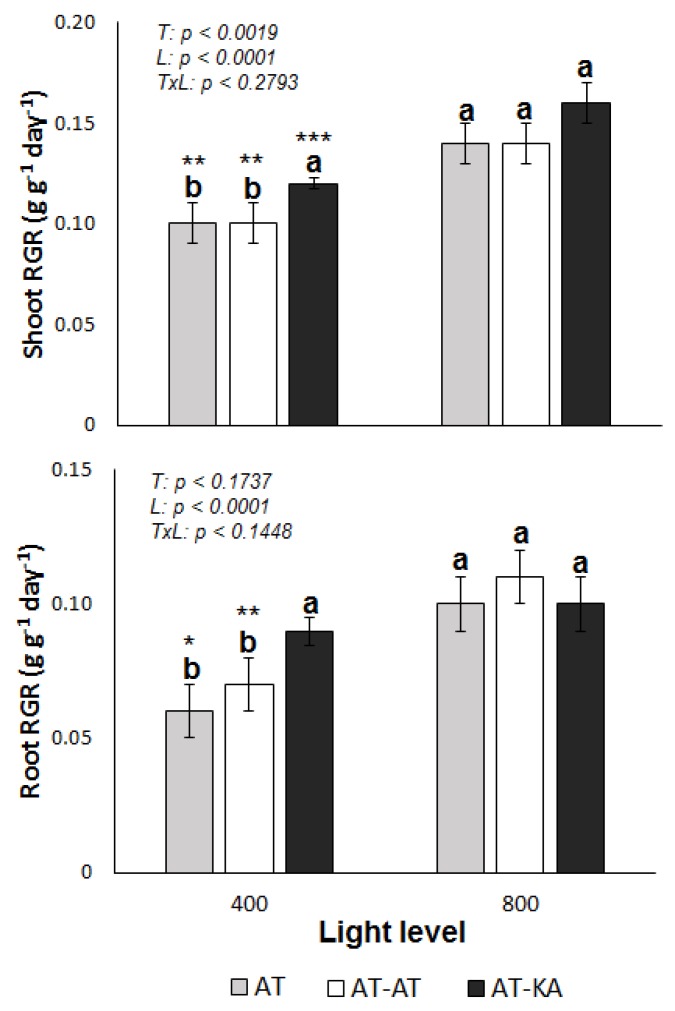
Shoot (**top**) and root (**bottom**) relative growth rate (RGR) in each treatment under medium (400 µmol m^−2^ s^−1^) or high (800 µmol m^−2^ s^−1^) light levels during a 30-day growth period. Bars represent mean ± SE of eight replicates. *p*-Values for treatment (*T*), light (*L*) and the interaction (*TxL*) effects are included in each case. Different letters denote significant differences (*p* < 0.05) among treatments within each light level. Asterisks indicate the significance level (*: *p* < 0.05; **: *p* < 0.01; ***: *p* < 0.0001) for the differences in the same treatment across light levels. AT: Non-grafted treatment; AT-AT: Homograft; and, AT-KA: Graft treatment.

**Figure 2:**
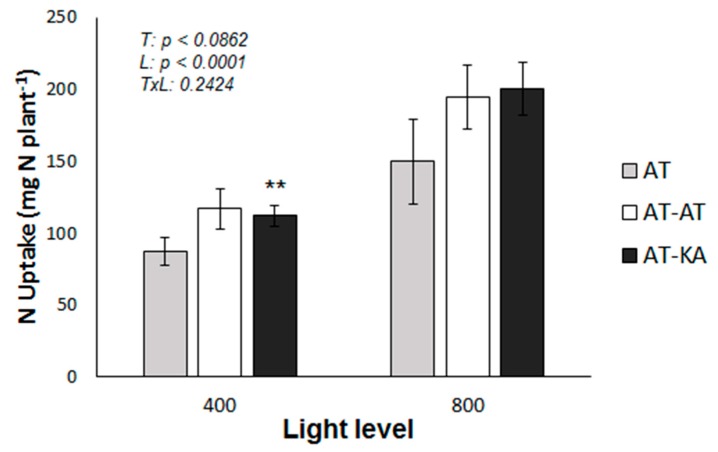
Total plant N uptake in each treatment under medium (400 µmol m^−2^ s^−1^) or high (800 µmol m^−2^ s^−1^) radiation during a 30-day period. Bars represent mean ± SE of eight replicates. *p*-Values for treatment (*T*), light (*L*) and the interaction (*TxL*) effects are included. Asterisks indicate the significance level (**: p < 0.01) for the differences in the same treatment across light levels. AT: Non-grafted treatment; AT-AT: Homograft; and, AT-KA: Graft treatment.

Shoot (*p* < 0.0001) and root relative growth rates (RGR) (*p* < 0.0001) were significantly affected by the light level in all treatments (Figure 1). Differences in shoot RGR among treatments were found only under medium light levels, where the graft treatment (AT-KA) showed higher values (*p* < 0.0017). Similar results were found from the analysis of root growth, where AT-KA showed higher RGR values than the control treatment (AT) under medium light intensity (*p* < 0.0121) (Figure 2). No differences in root RGR (*p* < 0.0881) were found in the AT-KA treatment between medium and high light (Figure 1). 

Grafting significantly reduced shoot N content (*p* < 0.0296) under medium light intensity, whereas no differences were found under high radiation (*p* < 0.1211) (Table 1). Root N content was not affected by the treatments (*p* < 0.6704) or light level (*p* < 0.2836), with an average value of 43.1 ± 2.1 mg N g^−1^ dry weight (DW).

When plant N uptake (PNU) was evaluated, no differences were found between the treatments under medium (*p* < 0.1828) or high (*p* < 0.2769) light intensity (Figure 2). However, increasing the radiation level significantly increased PNU in the AT-KA treatments (*p* < 0.0005) (Figure 2).

### 2.2. NO_3_^−^ Transporters Expression

**Figure 3:**
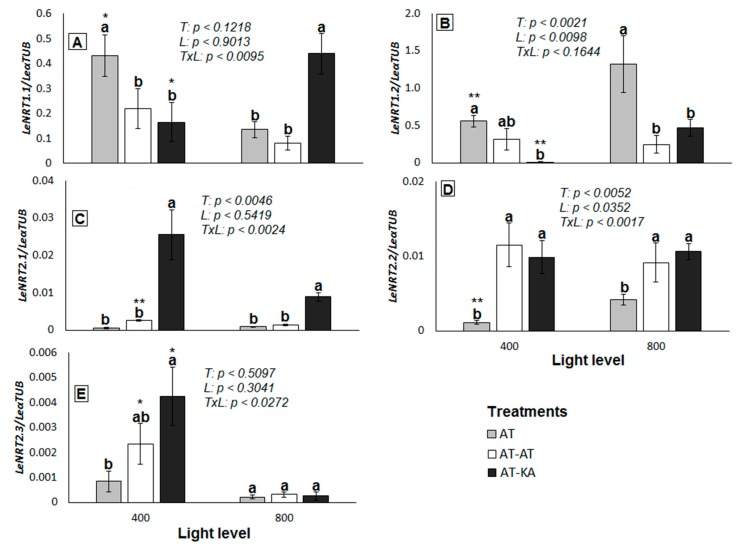
Relative expression of *LeNRT1.1* (**A**), *LeNRT1.2* (**B**), *LeNRT2.1* (**C**), *LeNRT2.2* (**D**), and *LeNRT2.3* (**E**) in roots of plants from each grafting combination exposed to medium (400 µmol PAR m^−2^ s^−1^) or high light level (800 µmol PAR m^−2^ s^−1^). The reference gene corresponds to α-tubulin. Bars represent mean ± SE of four replicates. *p*-Values for treatment (*T*), light (*L*) and the interaction (*TxL*) effects are included in each case. Different letters denote significant differences (*p* < 0.05) among treatments within each light level. Asterisks indicate the significance level (*: *p* < 0.05; **: *p* < 0.01) for the differences in the same treatment across light levels. AT: Non-grafted treatment; AT-AT: Homograft; and, AT-KA: Graft treatment.

The expression of genes from the NRT1 family was higher in all treatments in comparison to the NRT2 family (Figure 3). Both *LeNRT1.1* (*p* < 0.0467) and *LeNRT1.2* (*p* < 0.0069) increased their expression in the AT-KA treatment when switching from medium to high light intensity (Figure 3A,B). On the contrary, *LeNRT1.1* decreased its expression in the ungrafted plants under high light intensity (*p* < 0.0157) in comparison to the medium level of radiation (Figure 3A), whereas *LeNRT1.2* increased its expression to a level higher than in AT-KA (*p* < 0.0096) (Figure 3B). A higher expression of *LeNRT2.1* was found in AT-KA under medium (*p* < 0.0021) and high (*p* < 0.0242) light intensity in comparison to the non-grafted plants, where the expression of this gene remained unaltered independently of light intensity (Figure 3C). Something similar occurred with the expression of *LeNRT2.2*, which was higher in AT-KA than in the control treatment (*p* < 0.0352) (Figure 3D), although in this case, no differences in the expression level were found between the two light treatments (*p* < 0.7769). The high-affinity *LeNRT2.3* transporter showed the lowest level of expression from all the genes analyzed. Its expression was higher in the AT-KA treatment (*p* < 0.0468) in comparison to the control plants under medium light intensity (Figure 3E). No differences in the expression of this gene were found among the treatments under high radiation levels (*p* < 0.6120).

### 2.3. NO_3_^−^ Uptake Kinetic Parameters

No differences in *V*_max_ were found among treatments (*p* < 0.4787) but higher light intensities significantly increased this parameter in AT-KA (*p* < 0.0491) (Table 2). The apparent affinity for NO_3_^−^ uptake, represented by lower *K*_m_ values, increased in all treatments at higher light intensities (*p* < 0.002). No differences in *K*_m_ were found among the treatments (*p* < 0.2688).

### 2.4. Nitrate Reductase Activity

**Figure 4:**
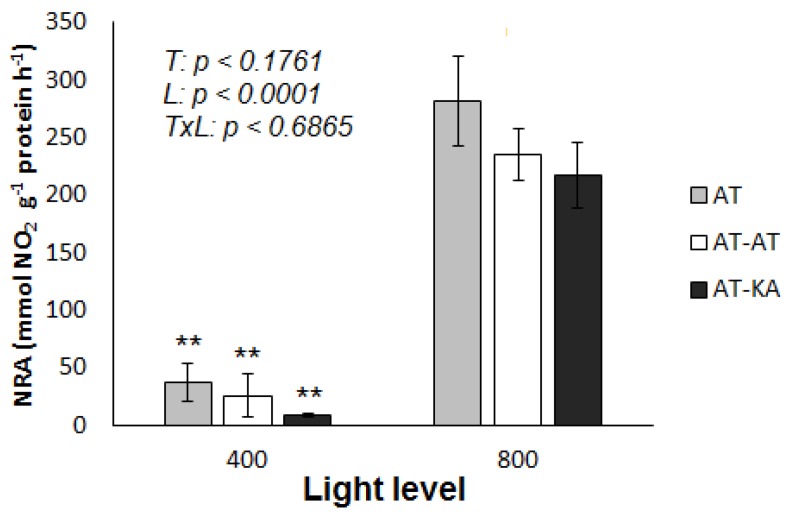
Nitrate Reductase Activity (NRA) in leaves from plants exposed to medium (400 µmol PAR m^−2^ s^−1^) or high (800 µmol PAR m^−2^ s^−1^) light level. Bars represent mean ± SE of four replicates. *p-Values* for treatment (*T*), light (*L*) and the interaction (*TxL*) effects are included. Asterisks indicate the significance level (**: *p* < 0.01) for the differences in the same treatment across light levels. AT: Non-grafted treatment; AT-AT: Homograft; and, AT-KA: Graft treatment.

No differences in nitrate reductase activity (NRA) between treatments were found under medium (*p* < 0.4239) or high (*p* < 0.3461) light intensity, with average values of 23.28 ± 8.40 and 244.46 ± 18.01 mmol NO_2_ g^−1^ protein h^−1^. However, all treatments showed a significant increase (*p* < 0.0001) when the light level was increased (Figure 4). No NRA was detected in the roots of any treatment under either light intensity.

### 2.5. Root Respiration

**Figure 5:**
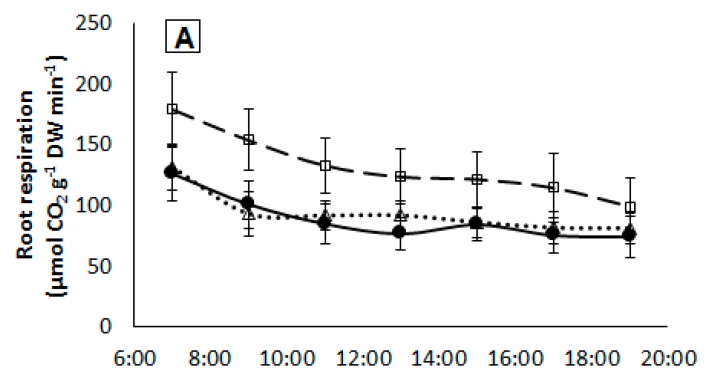

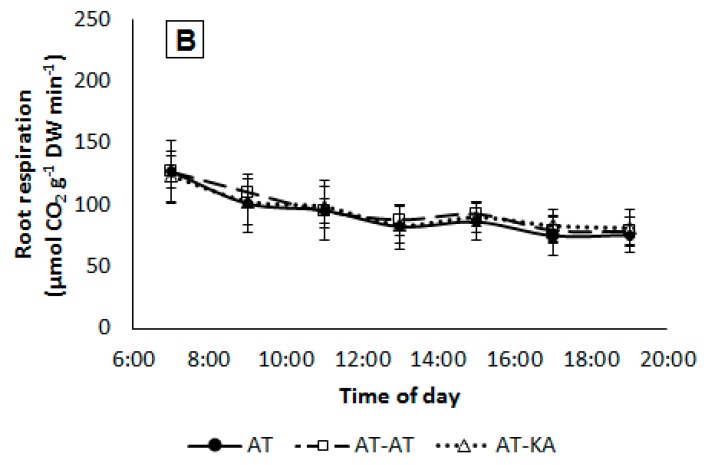
Daily time-course of root respiration in plants from each treatment growing under medium (**A**) or high (**B**) light intensity. Measurements started one hour before the lights turned on until one hour after the lights turned off. Symbols represent mean ± SE of eight replicates. AT: Non-grafted treatment; AT-AT: Homograft; and, AT-KA: Graft treatment.

Daily root respiration rates were similar in all treatments (*p* < 0.4037) with an average value of 157.8 ± 16.2 and 140.2 ± 12.2 mmol CO_2_ g^−1^ DW day^−1^ under medium and high light intensity, respectively. Hourly respiration rates showed a similar pattern in the AT-KA and control treatments, showing similar values under both light intensities (Figure 5).

## 3. Discussion

Total PNU increased by 71% in AT, 66% in AT-AT, and 93% in AT-KA when light increased from 400 to 800 µmol m^−2^ s^−1^. This increase in the radiation level has no effect on tissue N content, except in AT-KA where a 25.6% increase (*p* < 0.0014) in root N content occurred. This improvement in NO_3_^−^ absorption is mediated by a higher expression of both *LeNRT1.1* and *LeNRT1.2*, in the vigorous rootstock, whereas non-grafted plants rely almost exclusively on the expression of *LeNRT1.2*. These results contrast those reported for cucumber (*Cucumis sativus*) or tobacco (*Nicotiana tabacum*) where *NRT1.1* presents a higher expression than *NRT1.2* across various NO_3_^−^ supply [13,14]. 

Both transporters, *LeNRT1.1* and *LeNRT1.2*, act as symporters coupled to the uptake of H^+^ into the roots [7]. However, *LeNRT1.1* presents the capacity to absorb NO_3_^−^ in a wide range of external concentrations due to its ability to modify the protein structural flexibility by phosphorylation/dephosphorylation [15,16]. The only report available in tomatoes, showing no modification in the expression of *LeNRT1.1* under different growth conditions, relates this response to the colonization by mycorrhiza; the expression of *LeNRT2.3* is affected instead [17].

The increase in the expression of *LeNRT1.1* and *LeNRT1.2* in the rootstock is accompanied by a substantial decrease in the expression of *LeNRT2.1* and *LeNRT2.3*; *LeNRT2.1* encodes for a high-affinity transporter acting at low external NO_3_^−^ concentrations [7]. On the other hand, Fu et al. suggest that *LeNRT2.3* functions as a low-affinity transporter, involved in NO_3_^−^ loading into the xylem [6]. This activity would allow higher N-use efficiency in tomatoes [6,18], but our results show that rootstocks repress the synthesis of this transporter when shoot N-demand increases. 

The classic approach to studying NO_3_^−^ transporters expression is by manipulating NO_3_^−^ availability in the root zone [19,20,21,22]. More recently, a systems approach has been used to evaluate the coordination between nutrient uptake and environmental signals, such as carbon dioxide and light [23,24]. These studies have been conducted mainly on *Arabidopsis thaliana*, and no reports were found relating growth rate to the expression of NO_3_^−^ transporters in tomato roots. Here we show that the expression patterns of NO_3_^−^ transporters in the roots of tomato differ from those found in the rootstocks.

Available kinetics studies in the literature present V_max_ values in the range of 0.5–2.5 mmol g^−1^ DW h^−1^ [25,26]. Our results show higher uptake capacity, possibly because of the age of the plants used in our study, in comparison to the seedlings used by the previous authors. Although, no differences were found in the uptake capacity between the treatments (Table 2), the higher RGR found in the grafted plants suggests an enhancement in N utilization efficiency in the grafted treatments.

Despite the differences in the NO_3_^−^ uptake rates between medium and high light intensity, no differences in root respiration rates were found in AT-KA. Similar root respiration rates are reported in the literature for the tomato [27,28,29], but no information is available about the components of root respiration in this crop. In *Prunus* spp., Toro et al. [30] reported that rootstocks with higher growth rates present elevated root respiration rates, which is attributed exclusively to differences in the energy invested for root growth, not for nutrient uptake. Considering that NO_3_^−^ uptake and assimilation accounts for about 25% of the energy budget in roots [31], it is possible to assume that our findings in NO_3_^−^ uptake will not allow us to measure significantly different root respiration rates. Moreover, our results show that NO_3_^−^ is exclusively assimilated in the shoot, reducing the energy consumption in the roots from 15 ATP to only 1 ATP per mole of NO_3_^−^ absorbed because of the null activity of the enzymes involved in the assimilation process.

Among the processes controlling nutrient uptake rates, the assimilatory capacity has been suggested as a target for improving nutrient acquisition in crops [32,33]. However, our results show no difference in leaf NRA between the treatments, as reported for other crops like watermelon grafted onto pumpkin rootstocks [34]. No differences in the organ of assimilation were found either, which implies a higher accumulation of NO_3_^−^ in the roots of AT-KA when exposed to high light intensities.

## 4. Materials and Methods

### 4.1. Plant Material and Growth Conditions

Tomato (*Solanum lycopersicum* L. cv. Attiya, Rijk Zwan, De Lier, The Netherlands) plants and a vigorous interspecific hybrid (*S. lycopersicum* x *S. habrochaites* cv. Kaiser, Rijk Zwan, De Lier, The Netherlands) were obtained from seeds germinated in plastic trays placed in a growth chamber set at 25 °C air temperature. Plants were grafted at the two true leaves stage into one of these two combinations: Attiya self-grafted (AT-AT) or Attiya grafted onto Kaiser (AT-KA). A third treatment, corresponding to ungrafted Attiya (AT) plants, was used as a control. After callus formation, eight plants of each treatment were placed in a water culture system, containing 7.0 mM N-NO_3_, 3.0 mM K, 0.5 mM P, 2.0 mM Ca, 1.0 mM Mg and 1.0 mM S plus micronutrients. Plants were grown for 30 days under medium (400 µmol PAR m^−2^ s^−1^) or high (800 µmol PAR m^−2^ s^−1^) light intensity, using a 10 h photoperiod and 25/18 °C day/night air temperature. Within each light treatment, plants were arranged in a completely randomized design.

### 4.2. Growth Measurements and N Accumulation

At transplant, 10 plants of each treatment were harvested from the plastic trays, split into shoot and roots, and individually weighed. Later, plants were dried in an oven at 60 °C for 48 h to determine dry weight. The plants in the water culture were harvested 30 days after the experiment start and were weighed similarly as described before. Then, shoot and root relative growth rates (RGR, g g^−1^ day^−1^) were determined using the following equation:

RGR = ln(*DW_2_*) − ln(*DW_1_*)/*t*, (2)

where *DW_1_* and *DW_2_* represent dry weight, in g plant^−1^, at time 1 and time 2, respectively, whereas *t* corresponds to the growth period (30 days). After the dry weight was recorded, the N content in shoots and roots was determined by Kjeldhal distillation. Then, plant N uptake (PNU, mg N plant^−1^) was determined as follows:

PNU = [(*DW_S2_* × *N_S2_*) + (*DW_R2_* × *N_R2_*) − (*DW_S1_* × *N_S1_*) − (*DW_R1_* × *N_R1_*)]/100 (3)

where *DW_s_* and *DW_R_* represent shoot and root dry weight (g plant^−1^), respectively, while *N_S_* and *N_R_* are the shoot and root N content (%), respectively. Subscripts *1* and *2* denote the time of measurement.

### 4.3. Gene Expression

At harvest, four biological root samples from four different plants of each treatment were collected. Samples were immediately frozen at ‒80 °C until RNA extraction. Total RNA was extracted from 100 mg samples using RNA-Solv reagent and RNA quality was tested using electrophoresis in a 2% agarose gel stained with GelRed. Ribonucleic acid quantification was determined by spectrophotometry (model NanoDrop 2000, Thermo Scientific, Waltham, MA, USA). First strand cDNA synthesis was obtained from 1 µg of total RNA using SuperScript II reverse transcriptase (Invitrogen, Carlsbad, CA, USA) and random primers. Real-Time PCR reactions were carried out using four biological and two technical replicates for each target gene (*LeNRT1.1*, *LeNRT1.2*, *LeNRT2.1*, *LeNRT2.2* and *LeNRT2.3*). The PCR reactions were performed using gene-specific primers [31] (Table 3) and PowerUp SYBRgreen master mix (Applied Biosystems, Foster City, CA, USA). The reaction was run through a Real-Time PCR System (model StepOne, Thermo Fisher, Waltham, MA, USA) programmed at 94 °C for 1 min, followed by 35 cycles at 94 °C for 1 min, 55 °C for 1 min, and 72 °C for 1 min. Relative quantification was determined using α-tubulin as a reference gene [35]. 

### 4.4. Enzyme Activity

Nitrate reductase activity was determined in four biological leaf and root samples collected at harvest, according to the methodology described by Kaiser and Lewis [36] and modified by Reguera et al. [37]. One milliliter of extraction buffer (50 mM KH_2_PO_4_-KOH, pH 7.5, 2 mM EDTA, 2 mM dithiothreitol and 1% polyvinylpolypyrrolidone) was added to 0.1 g of frozen tissue and extracts were centrifuged at 20,000 g for 20 min at 4 °C. Then, 700 µL of reaction buffer (50 mM KH_2_PO_4_-KOH, pH 7.5, 10 mM KNO_3_ and 0.1 mM NADH) were added to 100 µL of total soluble proteins, and samples were incubated at 28 °C for 15 min. The reaction was stopped with the addition of 1 mL 1% sulphanilamide in 1.5 M HCl and 1 mL 0.02% n-l-naphtyl-ethylenediamine dihydrochloride. After 30 min, samples were centrifuged at 500 g for 5 min to remove suspended matter and nitrite was determined by absorbance at 540 nm in a spectrophotometer (model BioTek Power Wave HT, Shimadzu, Tokyo, Japan). Duplicate aliquots of extract from each sample replicate were assayed. Protein content was determined by Bradford’s assay (Coomassie Plus kit, Thermo Fisher, Waltham, MA, USA). 

### 4.5. NO_3_^−^ Uptake Kinetic Parameters

Root NO_3_^−^ uptake kinetic parameters (*V*_max_ and *K*_m_) were determined in a set of depletion experiments. Eight plants of each treatment were placed individually in 1-L containers and randomly arranged within each of the light conditions described above. Plants were allowed to grow for 30 days as in the experiment described above, and then roots were exposed consecutively to nutrient solutions containing 0.25, 0.5, 0.75, 1.0, 2.0, and 4.0 mM of NO_3_^−^. Five-milliliter samples were collected every 15 min in a six-hour period, and NO_3_^−^ content was determined by ion chromatography (model Dionex Aquion, Thermo Scientific) equipped with an anion pre-column (Dionex IonPac AG11-HC, 4 mm) and a separator column (Dionex IonPac AS11-HC, 4 mm) coupled with a self-regenerating suppressor (AERS 500, 4 mm). The eluent (30 mM KOH) was injected at a flow rate of 1 mL min^−1^. Nitrate uptake (U) was calculated as the difference between NO_3_^−^ content in the sample at the beginning of the experiment minus the content at the time when the concentration reached a steady state (usually between 120 and 240 min). The influx data were fitted to the Michaelis‒Menten relation (Equation (1)). Both *U* and *V*_max_ are expressed in mmol NO_3_^−^ g^−1^ DW h^−1^, while *K*_m_ and *C* are in mM.

### 4.6. Root Respiration

**Figure 6:**
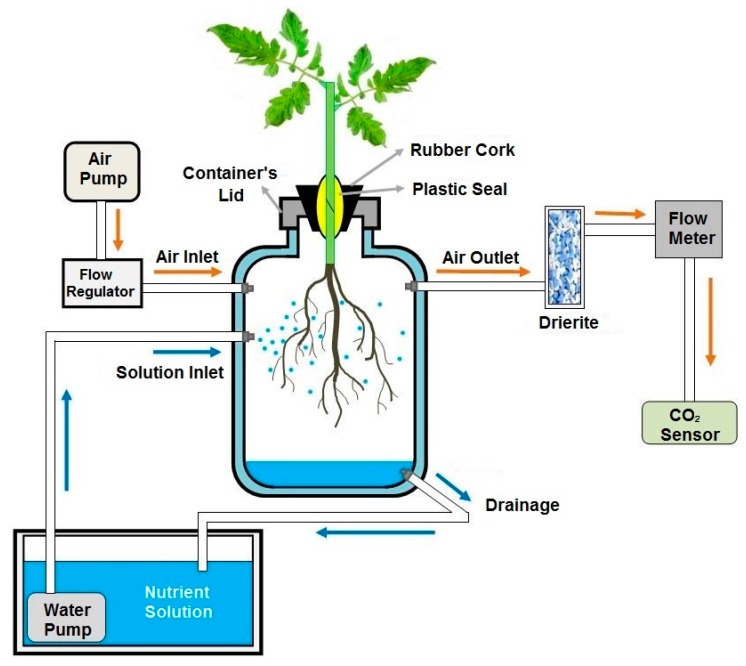
Diagram of the system used to measure root respiration rates.

In a separate set of plants, root respiration was determined using an open gas-exchange system. Four plants of each treatment were placed individually in 1-L plastic containers and randomly distributed in one of the light conditions described for the experiments detailed before. Each container was connected to a pump that continuously sprayed a nutrient solution onto the roots from a reservoir. The height of the solution within the containers was kept as low as possible by placing a drainage tube that lets out into the reservoir (Figure 6). The composition of the nutrient solution was similar to that described for the previous experiments but at a 50% dilution. A small hole was drilled in the container’s lid to allow plant suspension into the container. All connections and the lid of the container were sealed to avoid air leaks. Air was injected into each container at a 200 mL min^−1^ rate, and the CO_2_ concentration was monitored for two consecutive days from one hour before lights were turned on until one hour after lights were turned off (a 12-h period). This was repeated with four different sets of plants to build a dataset composed of eight replicates per treatment at each hour of measurement. The concentration of CO_2_ in the air was determined using a CO_2_ sensor (model QS151, Qubit) and root respiration rates were calculated as the difference between the CO_2_ content measured after passing through the container minus the content measured prior to entering the container. Results are expressed on a dry weight basis.

### 4.7. Statistical Analysis

Differences in growth rate, N content, gene expression, NRA, as well as daily and hourly root respiration rates were analyzed by ANOVA with mean separation by Tukey´s test. Kinetic parameters (*V*_max_ and *K*_m_) were estimated by non-linear regression analysis fitting a Michaelis‒Menten curve to the influx and concentration data. Estimated parameters were then analyzed by ANOVA to test the differences between treatments and light intensity. All analyses were conducted using R statistical software [38] through the InfoStat console [39].

## 5. Conclusions

The expression of the genes encoding for NO_3_^−^ transporters in tomato roots differs from the expression of these genes in the roots of a vigorous rootstock. When shoot N-demand is high, rootstocks present a similar level of expression for *LeNRT1.1* and *LeNRT1.2*, whereas in the non-grafted plants, *LeNRT1.2* is the exclusive transporter with the highest expression. Grafting tomatoes onto vigorous rootstocks does not affect the organ of NO_3_^−^ assimilation nor the activity of nitrate reductase. 

## Figures and Tables

**Table 1:** Tissue N content in each treatment under medium or high light intensity. Data are means of four biological independent replicates ± SE. Different letters in the same column denote significant differences (*p* < 0.05). AT: Non-grafted treatment; AT-AT: Homograft; and, AT-KA: Graft treatment.

Treatment	Tissue N Content (mg g^−1^ DW)			
	400 µmol m^−2^ s^−1^		800 µmol m^−2^ s^−1^	
	**Shoot**	**Root**	**Shoot**	**Root**
AT	59.3 ± 0.7 a	45.1 ± 3.1 a	59.0 ± 1.1 a	38.7 ± 3.6 a
AT-AT	57.0 ± 0.1 b	42.5 ± 2.1 a	58.6 ± 0.9 a	45.4 ± 1.4 a
AT-KA	55.4 ± 0.3 b	38.4 ± 1.6 a	57.6 ± 1.0 a	48.3 ± 0.7 a

**Table 2:** Apparent *V*_max_ and *K*_m_ of net nitrate uptake for each treatment under medium (400 µmol PAR m^−2^ s^−1^) or high (800 µmol PAR m^−2^ s^−1^) light intensity. Data are means of eight biological independent replicates ± SE. AT: Non-grafted treatment; AT-AT: Homograft; and, AT-KA: Graft treatment.

Treatment	*V*_max_ (mmol g^−1^ DW h^−1^)		*K*_m_ (mM)	
	400	800	400	800
AT	5.56 ± 1.05	7.53 ± 1.36	0.16 ± 0.02	0.04 ± 0.02
AT-AT	6.37 ± 1.33	7.20 ± 1.65	0.35 ± 0.10	0.02 ± 0.01
AT-KA	3.34 ± 1.72	7.21 ± 0.50	0.17 ± 0.08	0.06 ± 0.04

**Table 3:** Gene-specific primers for RT-PCR (Yao et al., 2008).

Gene	Primer
*LeNRT1.1*	Forward: TACTATTCAAGCTATGGGTGTTACG
	Reverse: ATTTGTCCTCTTTCTTTTTTGTCCG
*LeNRT1.2*	Forward: TTTTAGGTGTTGAAGCTGTGGAGAG
	Reverse: GCGATGTATAGGACCATGAGTTGTT
*LeNRT2.1*	Forward: TTCCTGTTACATTTTGTCATTTCCC
	Reverse: CAGATTCAAGACTATCCATTCCTCA
*LeNRT2.2*	Forward: TCAAGGGAACGGAAGAACATTATTA
	Reverse: GCTCATTGAACTAAAGATTGACGAT
*LeNRT2.3*	Forward: AATGCATGGTGTTACTGGTAGAGAG
	Reverse: CTAATAATAGGGACTAAAGGGGCTG

## References

[1] Singh H., Kumar P., Chaudhari S., Edelstein M. (2017). Tomato grafting: A global perspective. HortScience.

[2] Lee J., Kubota C., Tsao S.J., Bie Z., Hoyos Echevarria P., Morra L., Oda M. (2010). Current status of vegetable grafting: Diffusion, grafting techniques, automation. Sci. Hortic..

[3] Djidonou D., Zhao X., Simonne E.H., Koch K.E., Erickson J.E. (2013). Yield, water-, and nitrogen-use efficiency in field-grown, grafted tomatoes. HortScience.

[4] Magalhaes J.S., Wilcox G.E. (1983). Tomato growth and nutrient uptake patterns as influenced by nitrogen form and light intensity. J. Plant Nutr..

[5] Errebhi M., Wilcox G.E. (1990). Tomato growth and nutrient uptake pattern as influenced by nitrogen form ratio. J. Plant Nutr..

[6] Fu Y., Yi H., Bao J., Gong J. (2015). LeNRT2.3 functions in nitrate acquisition and long-distance transport in tomato. FEBS Lett..

[7] Ono F., Frommer W.B., Von Wiren N. (2000). Coordinated diurnal regulation of low- and high-affinity nitrate transporters in tomato. Plant Biol..

[8] Le Deunff E., Tournier P., Malagoli P. (2016). The thermodynamic flow-force interpretation of root nutrient uptake kinetics: A powerful formalism for agronomic and phytoplanktonic models. Front. Physiol..

[9] Von Wiren N., Gazzarrini S., Frommer W.B. (1997). Regulation of mineral nitrogen uptake in plants. Plant Soil.

[10] Gent L., Forde B.G. (2017). How do plants sense their nitrogen status?. J. Exp. Bot..

[11] Le Bot J., Jeannequin B., Fabre R. (2001). Growth and nitrogen status of soilless tomato plants following nitrate withdrawal from the nutrient solution. Ann. Bot..

[12] Imsande J., Touraine B. (1994). N demand and the regulation of nitrate uptake. Plant Physiol..

[13] Migoka M., Warzybok A., Klobus G. (2013). The genomic organization and transcriptional pattern of genes encoding nitrate transporters 1 (NRT1) in cucumber. Plant Soil.

[14] Liu L., Fan T., Shi D., Li C., He M., Chen Y., Zhang L., Yang C., Cheng X., Chen X. (2018). Coding-sequence identification and transcriptional profiling of nine AMTs and four NRTs from tobacco revealed their differential regulation by developmental stages, nitrogen nutrition, and photoperiod. Front. Plant Sci..

[15] Sun J., Zheng N. (2015). Molecular mechanism underlying the plant NRT1.1 dual-affinity nitrate transporter. Front. Physiol..

[16] Rashid M., Bera S., Medvinsky A.B., Sun G., Li B., Chakraborty A. (2018). Adaptive regulation of nitrate transceptor NRT1.1 in fluctuating soil nitrate conditions. iScience.

[17] Hildebrandt U., Schmelzer E., Bothe H. (2002). Expression of nitrate transporter genes in tomato colonized by an arbuscular mycorrhizal fungus. Physiol. Plantarum.

[18] Fan X., Naz M., Fan X., Xuan W., Miller A.J., Xu G. (2017). Plant nitrate transporters: From gene function to application. J. Exp. Bot..

[19] Wang Y., Garvin D.F., Kochian L.V. (2001). Nitrate-induced genes in tomato roots. Array analysis reveals novel genes that may play a role in nitrogen nutrition. Plant Physiol..

[20] Little D.Y., Rao H., Oliva S., Daniel-Vedele F., Krapp A., Malamy J.E. (2005). The putative high-affinity nitrate transporter NRT2.1 represses lateral root initiation in response to nutritional cues. Proc. Natl. Acad. Sci. USA.

[21] Tsay Y., Chiu C., Tsai C., Ho C., Hsu P. (2007). Nitrate transporters and peptide transporters. FEBS Lett..

[22] Krouk G., Crawford N.M., Coruzzi G.M., Tsay Y. (2010). Nitrate signaling: Adaptation to fluctuating environemnts. Curr. Opin. Plant Biol..

[23] Krouk G., Tranchina D., Lejay L., Cruikshank A.A., Shasha D., Coruzzi G.M., Gutierrez R.A. (2009). A systems approach uncovers restrictions for signal interactions regulating genome-wide responses to nutritional cues in *Arabidopsis*. PLOS Comput. Biol..

[24] Alvarez J.M., Riveras E., Vidal E.A., Gras D.E., Contreras-Lopez O., Tamayo K.P., Aceituno F., Gomez I., Ruffel S., Lejay L. (2014). Systems approach identifies TGA1 and TGA4 transcription factors as important regulatory components of the nitrate response of *Arabidopsis thaliana* roots. Plant J..

[25] Smart D.R., Bloom A.J. (1988). Kinetics of ammonium and nitrate uptake among wild and cultivated tomatoes. Oecologia.

[26] Cardenas-Navarro R., Adamowicz S., Gojon A., Robin P. (1999). Modelling nitrate influx in young tomato (*Lycopersicon esculebtum* Mill.) plants. J. Exp. Bot..

[27] Klock K.A., Taber H.G., Graves W.R. (1997). Root respiration and phosphorus nutrition of tomato plants grown at a 36°C root-zone temperature. J. Am. Soc. Hortic. Sci..

[28] Shi K., Hu W., Dong D., Zhou Y., Yu J. (2007). Low O_2_ supply is involved in the poor growth in root-restricted plants of tomato (*Lycopersicon esculentum* Mill.). Environ. Exp. Bot..

[29] Horchani F., Khayati H., Raymond P., Brouquisse R., Aschi-Smiti S. (2009). Contrasted effects of prolonged root hypoxia on tomato root and fruit (*Solanum lycopersicum*) metabolism. J. Agron. Crop Sci..

[30] Toro G., Pinto M., Pimentel P. (2018). Root respiratory components of *Prunus spp.* Rootstocks under low oxygen: Regulation of growth, maintenance, and ion uptake respiration. Sci. Hortic..

[31] Taiz L., Zeiger E. (2006). Plant Physiology.

[32] Garnet T., Conn V., Kaiser B.N. (2009). Root based approaches to improving nitrogen use efficiency in plants. Plant Cell Environ..

[33] Loussaert D., Clapp J., Mongar N., O’Neill D.P., Shen B. (2018). Nitrate assimilation limits nitrogen use efficiency (NUE) in maize (*Zea mays* L.). Agronomy.

[34] Azher M., Wang L., Jiao Y., Chen C., Zhao L., Mei M., Yu Y., Bie Z., Huang Y. (2017). Pumpkin rootstock improves nitrogen use efficiency of watermelon scion by enhancing nutrient uptake, cytokinin content, and expression of nitrate reductase genes. Plant Growth Regul..

[35] Yao J., Shi W.M., Xu W.F. (2008). Effects of salt stress on expression of nitrate transporter and assimilation-related genes in tomato roots. Russ. J. Plant Physiol..

[36] Kaiser J., Lewis O. (1984). Nitrate reductase and glutamine synthetase activity in leaves and roots of nitrate-fed *Helianthus annuus* L.. Plant Soil.

[37] Reguera M., Peleg Z., Abdel-Tawab Y.M., Tumimbang E.B., Delatorre C.A., Blumwald E. (2013). Stress-induced cytokinin synthesis increases drought tolerance through the coordinated regulation of carbon and nitrogen assimilation in rice. Plant Physiol..

[38] R Development Core Team (2008). R: A Language and Environment for Statistical Computing.

[39] Di Rienzo J.A., Casanoves F., Balzarini M.G., Gonzalez L., Tablada M., Robledo C.W. (2014). InfoStat Version 2014 Grupo InfoStat.

